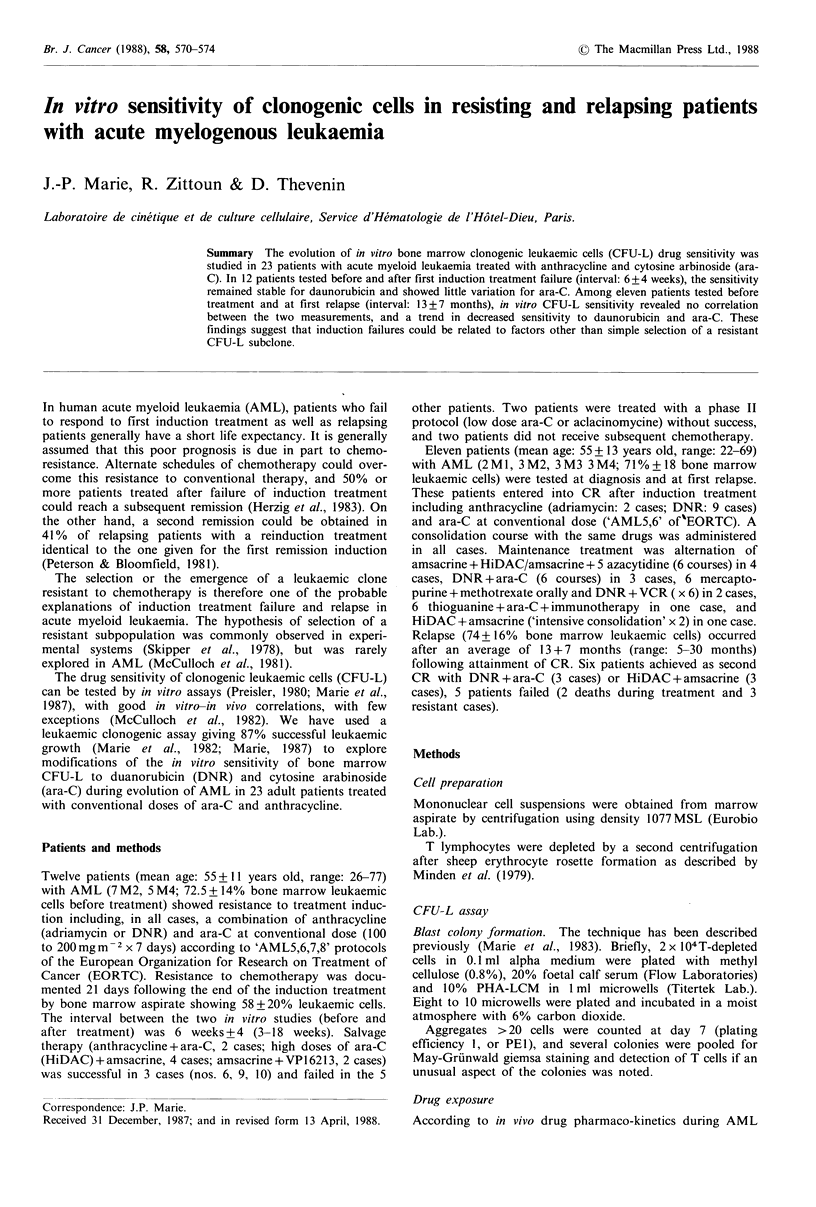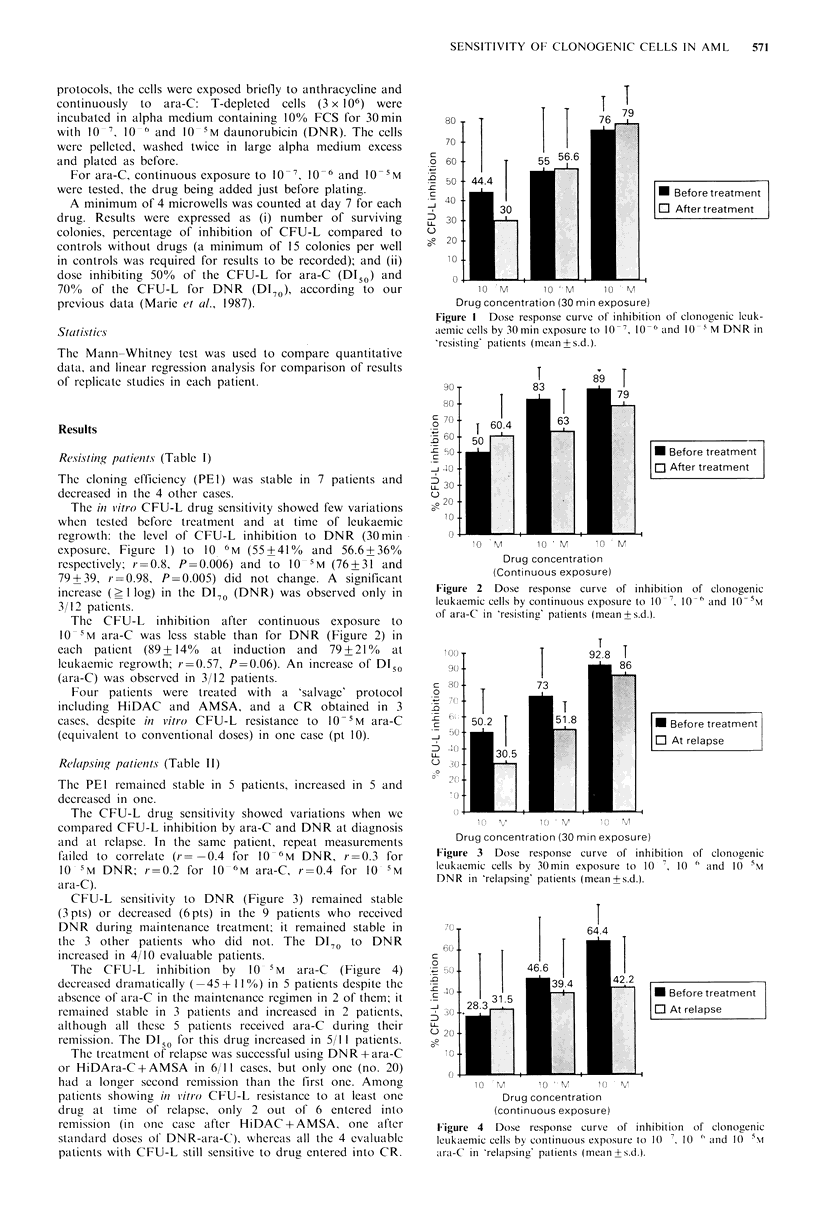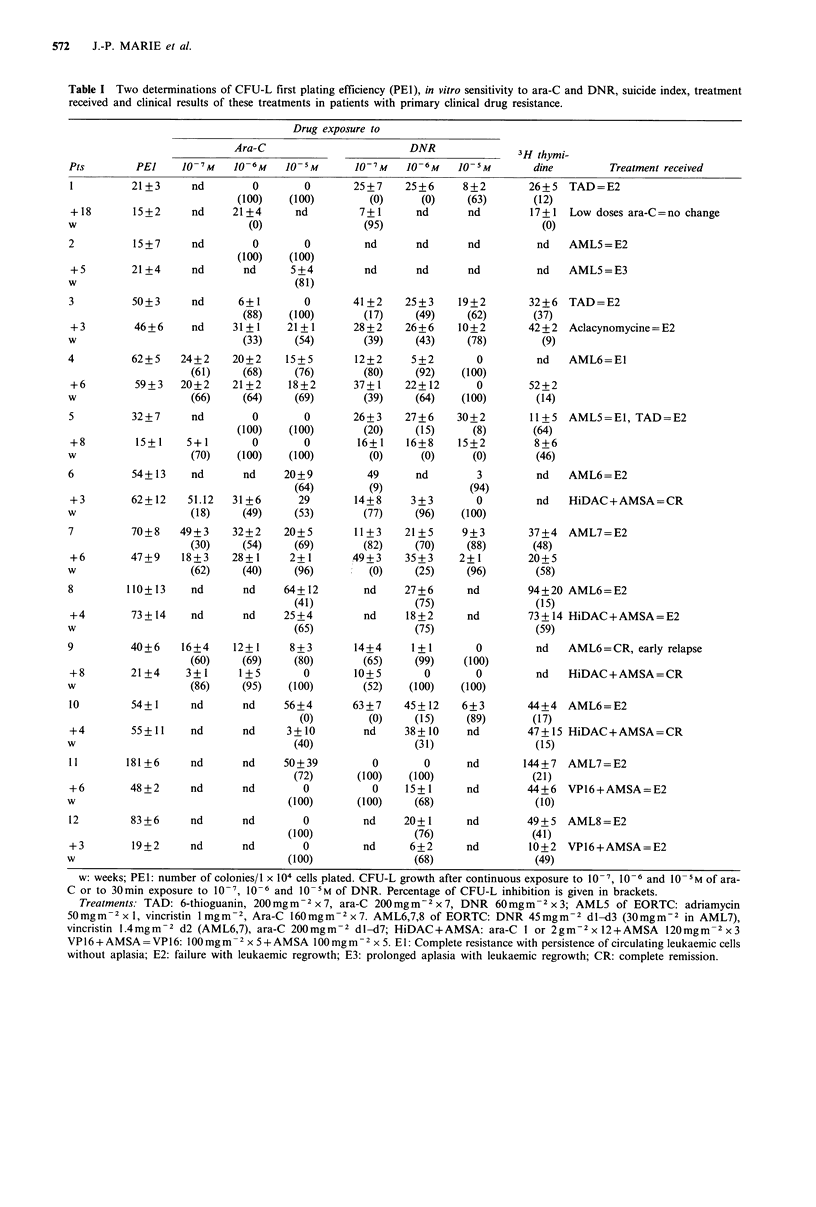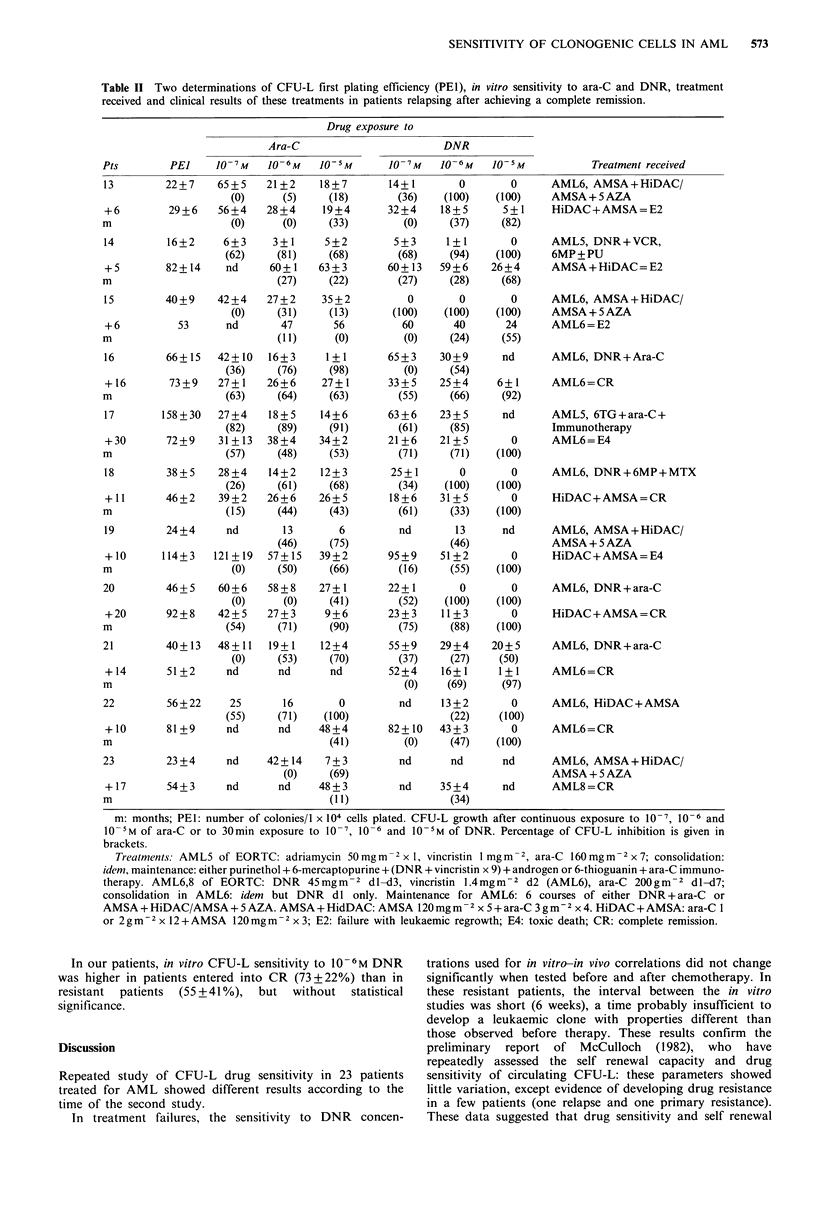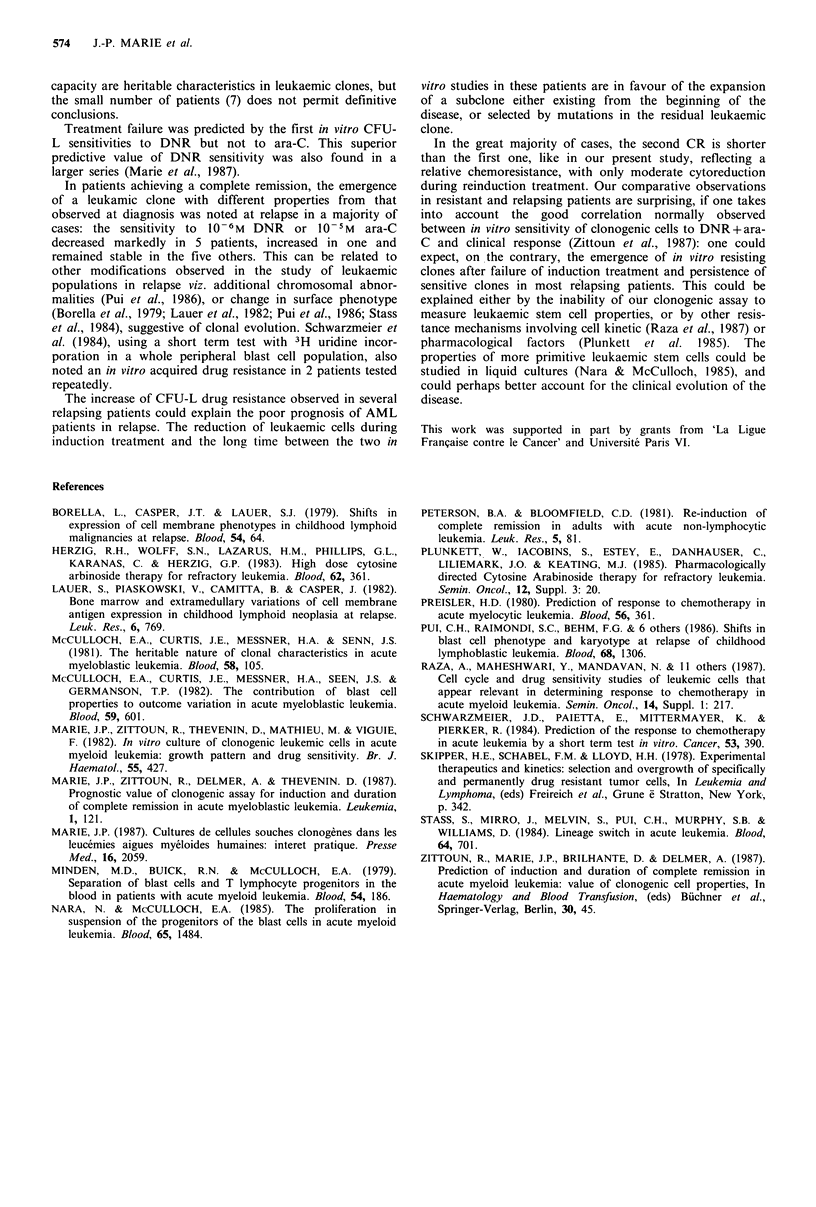# In vitro sensitivity of clonogenic cells in resisting and relapsing patients with acute myelogenous leukaemia.

**DOI:** 10.1038/bjc.1988.261

**Published:** 1988-11

**Authors:** J. P. Marie, R. Zittoun, D. Thevenin

**Affiliations:** Service d'HÃ¨matologie de l'HÃ´tel-Dieu, Paris.

## Abstract

The evolution of in vitro bone marrow clonogenic leukaemic cells (CFU-L) drug sensitivity was studied in 23 patients with acute myeloid leukaemia treated with anthracycline and cytosine arbinoside (ara-C). In 12 patients tested before and after first induction treatment failure (interval: 6 +/- 4 weeks), the sensitivity remained stable for daunorubicin and showed little variation for ara-C. Among eleven patients tested before treatment and at first relapse (interval: 13 +/- 7 months), in vitro CFU-L sensitivity revealed no correlation between the two measurements, and a trend in decreased sensitivity to daunorubicin and ara-C. These findings suggest that induction failures could be related to factors other than simple selection of a resistant CFU-L subclone.


*Br. J. Cancer* (1988), **58**, 570–574
© The Macmillan Press Ltd., 1988

### *In vitro* sensitivity of clonogenic cells in resisting and relapsing patients with acute myelogenous leukaemia

J.-P. Marie, R. Zittoun & D. Thevenin

*Laboratoire de cinétique et de culture cellulaire, Service d'Hématologie de l'Hôtel-Dieu, Paris.*

**Summary** The evolution of *in vitro* bone marrow clonogenic leukaemic cells (CFU-L) drug sensitivity was studied in 23 patients with acute myeloid leukaemia treated with anthracycline and cytosine arbinoside (ara-C). In 12 patients tested before and after first induction treatment failure (interval: $6 \pm 4$ weeks), the sensitivity remained stable for daunorubicin and showed little variation for ara-C. Among eleven patients tested before treatment and at first relapse (interval: $13 \pm 7$ months), *in vitro* CFU-L sensitivity revealed no correlation between the two measurements, and a trend in decreased sensitivity to daunorubicin and ara-C. These findings suggest that induction failures could be related to factors other than simple selection of a resistant CFU-L subclone.

In human acute myeloid leukaemia (AML), patients who fail to respond to first induction treatment as well as relapsing patients generally have a short life expectancy. It is generally assumed that this poor prognosis is due in part to chemo-resistance. Alternate schedules of chemotherapy could overcome this resistance to conventional therapy, and 50% or more patients treated after failure of induction treatment could reach a subsequent remission (Herzig *et al.*, 1983). On the other hand, a second remission could be obtained in 41% of relapsing patients with a reinduction treatment identical to the one given for the first remission induction (Peterson & Bloomfield, 1981).

The selection or the emergence of a leukaemic clone resistant to chemotherapy is therefore one of the probable explanations of induction treatment failure and relapse in acute myeloid leukaemia. The hypothesis of selection of a resistant subpopulation was commonly observed in experimental systems (Skipper *et al.*, 1978), but was rarely explored in AML (McCulloch *et al.*, 1981).

The drug sensitivity of clonogenic leukaemic cells (CFU-L) can be tested by *in vitro* assays (Preisler, 1980; Marie *et al.*, 1987), with good *in vitro–in vivo* correlations, with few exceptions (McCulloch *et al.*, 1982). We have used a leukaemic clonogenic assay giving 87% successful leukaemic growth (Marie *et al.*, 1982; Marie, 1987) to explore modifications of the *in vitro* sensitivity of bone marrow CFU-L to duanorubicin (DNR) and cytosine arabinoside (ara-C) during evolution of AML in 23 adult patients treated with conventional doses of ara-C and anthracycline.

### Patients and methods

Twelve patients (mean age: $55 \pm 11$ years old, range: 26–77) with AML (7 M2, 5 M4; $72.5 \pm 14\%$ bone marrow leukaemic cells before treatment) showed resistance to treatment induction including, in all cases, a combination of anthracycline (adriamycin or DNR) and ara-C at conventional dose (100 to 200 $mg\,m^{-2} \times 7$ days) according to 'AML5,6,7,8' protocols of the European Organization for Research on Treatment of Cancer (EORTC). Resistance to chemotherapy was documented 21 days following the end of the induction treatment by bone marrow aspirate showing $58 \pm 20\%$ leukaemic cells. The interval between the two *in vitro* studies (before and after treatment) was 6 weeks $\pm 4$ (3–18 weeks). Salvage therapy (anthracycline + ara-C, 2 cases; high doses of ara-C (HiDAC) + amsacrine, 4 cases; amsacrine + VP16213, 2 cases) was successful in 3 cases (nos. 6, 9, 10) and failed in the 5 other patients. Two patients were treated with a phase II protocol (low dose ara-C or aclacinomycine) without success, and two patients did not receive subsequent chemotherapy.

Eleven patients (mean age: $55 \pm 13$ years old, range: 22–69) with AML (2 M1, 3 M2, 3 M3 3 M4; $71\% \pm 18$ bone marrow leukaemic cells) were tested at diagnosis and at first relapse. These patients entered into CR after induction treatment including anthracycline (adriamycin: 2 cases; DNR: 9 cases) and ara-C at conventional dose ('AML5,6' of EORTC). A consolidation course with the same drugs was administered in all cases. Maintenance treatment was alternation of amsacrine + HiDAC/amsacrine + 5 azacytidine (6 courses) in 4 cases, DNR + ara-C (6 courses) in 3 cases, 6 mercaptopurine + methotrexate orally and DNR + VCR ( × 6) in 2 cases, 6 thioguanine + ara-C + immunotherapy in one case, and HiDAC + amsacrine ('intensive consolidation' × 2) in one case. Relapse ($74 \pm 16\%$ bone marrow leukaemic cells) occurred after an average of $13 + 7$ months (range: 5–30 months) following attainment of CR. Six patients achieved as second CR with DNR + ara-C (3 cases) or HiDAC + amsacrine (3 cases), 5 patients failed (2 deaths during treatment and 3 resistant cases).

### Methods

### *Cell preparation*

Mononuclear cell suspensions were obtained from marrow aspirate by centrifugation using density 1077 MSL (Eurobio Lab.).

T lymphocytes were depleted by a second centrifugation after sheep erythrocyte rosette formation as described by Minden *et al.* (1979).

### *CFU-L assay*

*Blast colony formation.* The technique has been described previously (Marie *et al.*, 1983). Briefly, $2 \times 10^4$ T-depleted cells in 0.1 ml alpha medium were plated with methyl cellulose (0.8%), 20% foetal calf serum (Flow Laboratories) and 10% PHA-LCM in 1 ml microwells (Titertek Lab.). Eight to 10 microwells were plated and incubated in a moist atmosphere with 6% carbon dioxide.

Aggregates >20 cells were counted at day 7 (plating efficiency 1, or PE1), and several colonies were pooled for May-Grünwald giemsa staining and detection of T cells if an unusual aspect of the colonies was noted.

### *Drug exposure*

According to *in vivo* drug pharmaco-kinetics during AML

Correspondence: J.P. Marie.
Received 31 December, 1987; and in revised form 13 April, 1988.

protocols, the cells were exposed briefly to anthracycline and continuously to ara-C: T-depleted cells ($3 \times 10^6$) were incubated in alpha medium containing 10% FCS for 30 min with $10^{-7}$, $10^{-6}$ and $10^{-5}$ M daunorubicin (DNR). The cells were pelleted, washed twice in large alpha medium excess and plated as before.

For ara-C, continuous exposure to $10^{-7}$, $10^{-6}$ and $10^{-5}$ M were tested, the drug being added just before plating.

A minimum of 4 microwells was counted at day 7 for each drug. Results were expressed as (i) number of surviving colonies, percentage of inhibition of CFU-L compared to controls without drugs (a minimum of 15 colonies per well in controls was required for results to be recorded); and (ii) dose inhibiting 50% of the CFU-L for ara-C ($DI_{50}$) and 70% of the CFU-L for DNR ($DI_{70}$), according to our previous data (Marie *et al.*, 1987).

*Statistics*

The Mann–Whitney test was used to compare quantitative data, and linear regression analysis for comparison of results of replicate studies in each patient.

### Results

*Resisting patients* (Table I)

The cloning efficiency (PEl) was stable in 7 patients and decreased in the 4 other cases.

The *in vitro* CFU-L drug sensitivity showed few variations when tested before treatment and at time of leukaemic regrowth: the level of CFU-L inhibition to DNR (30 min exposure, Figure 1) to $10^{-6}$ M ($55 \pm 41\%$ and $56.6 \pm 36\%$ respectively; $r = 0.8$, $P = 0.006$) and to $10^{-5}$ M ($76 \pm 31$ and $79 \pm 39$, $r = 0.98$, $P = 0.005$) did not change. A significant increase ($\geq 1$ log) in the $DI_{70}$ (DNR) was observed only in 3/12 patients.

The CFU-L inhibition after continuous exposure to $10^{-5}$ M ara-C was less stable than for DNR (Figure 2) in each patient ($89 \pm 14\%$ at induction and $79 \pm 21\%$ at leukaemic regrowth; $r = 0.57$, $P = 0.06$). An increase of $DI_{50}$ (ara-C) was observed in 3/12 patients.

Four patients were treated with a 'salvage' protocol including HiDAC and AMSA, and a CR obtained in 3 cases, despite *in vitro* CFU-L resistance to $10^{-5}$ M ara-C (equivalent to conventional doses) in one case (pt 10).

*Relapsing patients* (Table II)

The PEl remained stable in 5 patients, increased in 5 and decreased in one.

The CFU-L drug sensitivity showed variations when we compared CFU-L inhibition by ara-C and DNR at diagnosis and at relapse. In the same patient, repeat measurements failed to correlate ($r = -0.4$ for $10^{-6}$ M DNR, $r = 0.3$ for $10^{-5}$ M DNR; $r = 0.2$ for $10^{-6}$ M ara-C, $r = 0.4$ for $10^{-5}$ M ara-C).

CFU-L sensitivity to DNR (Figure 3) remained stable (3 pts) or decreased (6 pts) in the 9 patients who received DNR during maintenance treatment; it remained stable in the 3 other patients who did not. The $DI_{70}$ to DNR increased in 4/10 evaluable patients.

The CFU-L inhibition by $10^{-5}$ M ara-C (Figure 4) decreased dramatically ($-45 + 11\%$) in 5 patients despite the absence of ara-C in the maintenance regimen in 2 of them; it remained stable in 3 patients and increased in 2 patients, although all these 5 patients received ara-C during their remission. The $DI_{50}$ for this drug increased in 5/11 patients.

The treatment of relapse was successful using DNR + ara-C or HiDAra-C + AMSA in 6/11 cases, but only one (no. 20) had a longer second remission than the first one. Among patients showing *in vitro* CFU-L resistance to at least one drug at time of relapse, only 2 out of 6 entered into remission (in one case after HiDAC + AMSA, one after standard doses of DNR-ara-C), whereas all the 4 evaluable patients with CFU-L still sensitive to drug entered into CR.

**Figure 1** Dose response curve of inhibition of clonogenic leukaemic cells by 30 min exposure to $10^{-7}$, $10^{-6}$ and $10^{-5}$ M DNR in 'resisting' patients (mean ± s.d.).

**Figure 2** Dose response curve of inhibition of clonogenic leukaemic cells by continuous exposure to $10^{-7}$, $10^{-6}$ and $10^{-5}$ M of ara-C in 'resisting' patients (mean ± s.d.).

**Figure 3** Dose response curve of inhibition of clonogenic leukaemic cells by 30 min exposure to $10^{-7}$, $10^{-6}$ and $10^{-5}$ M DNR in 'relapsing' patients (mean ± s.d.).

**Figure 4** Dose response curve of inhibition of clonogenic leukaemic cells by continuous exposure to $10^{-7}$, $10^{-6}$ and $10^{-5}$ M ara-C in 'relapsing' patients (mean ± s.d.).

**Table I** Two determinations of CFU-L first plating efficiency (PE1), *in vitro* sensitivity to ara-C and DNR, suicide index, treatment received and clinical results of these treatments in patients with primary clinical drug resistance.

| | | *Drug exposure to* | | | | | | | |
|---|---|---|---|---|---|---|---|---|---|
| | | *Ara-C* | | | *DNR* | | | | |
| *Pts* | *PE1* | *$10^{-7}$M* | *$10^{-6}$M* | *$10^{-5}$M* | *$10^{-7}$M* | *$10^{-6}$M* | *$10^{-5}$M* | *$^{3}$H thymidine* | *Treatment received* |
| 1 | 21±3 | nd | 0<br>(100) | 0<br>(100) | 25±7<br>(0) | 25±6<br>(0) | 8±2<br>(63) | 26±5<br>(12) | TAD=E2 |
| +18<br>w | 15±2 | nd | 21±4<br>(0) | nd | 7±1<br>(95) | nd | nd | 17±1<br>(0) | Low doses ara-C=no change |
| 2 | 15±7 | nd | 0<br>(100) | 0<br>(100) | nd | nd | nd | nd | AML5=E2 |
| +5<br>w | 21±4 | nd | nd | 5±4<br>(81) | nd | nd | nd | nd | AML5=E3 |
| 3 | 50±3 | nd | 6±1<br>(88) | 0<br>(100) | 41±2<br>(17) | 25±3<br>(49) | 19±2<br>(62) | 32±6<br>(37) | TAD=E2 |
| +3<br>w | 46±6 | nd | 31±1<br>(33) | 21±1<br>(54) | 28±2<br>(39) | 26±6<br>(43) | 10±2<br>(78) | 42±2<br>(9) | Aclacynomycine=E2 |
| 4 | 62±5 | 24±2<br>(61) | 20±2<br>(68) | 15±5<br>(76) | 12±2<br>(80) | 5±2<br>(92) | 0<br>(100) | nd | AML6=E1 |
| +6<br>w | 59±3 | 20±2<br>(66) | 21±2<br>(64) | 18±2<br>(69) | 37±1<br>(39) | 22±12<br>(64) | 0<br>(100) | 52±2<br>(14) | |
| 5 | 32±7 | nd | 0<br>(100) | 0<br>(100) | 26±3<br>(20) | 27±6<br>(15) | 30±2<br>(8) | 11±5<br>(64) | AML5=E1, TAD=E2 |
| +8<br>w | 15±1 | 5+1<br>(70) | 0<br>(100) | 0<br>(100) | 16±1<br>(0) | 16±8<br>(0) | 15±2<br>(0) | 8±6<br>(46) | |
| 6 | 54±13 | nd | nd | 20±9<br>(64) | 49<br>(9) | nd | 3<br>(94) | nd | AML6=E2 |
| +3<br>w | 62±12 | 51.12<br>(18) | 31±6<br>(49) | 29<br>(53) | 14±8<br>(77) | 3±3<br>(96) | 0<br>(100) | nd | HiDAC+AMSA=CR |
| 7 | 70±8 | 49±3<br>(30) | 32±2<br>(54) | 20±5<br>(69) | 11±3<br>(82) | 21±5<br>(70) | 9±3<br>(88) | 37±4<br>(48) | AML7=E2 |
| +6<br>w | 47±9 | 18±3<br>(62) | 28±1<br>(40) | 2±1<br>(96) | 49±3<br>(0) | 35±3<br>(25) | 2±1<br>(96) | 20±5<br>(58) | |
| 8 | 110±13 | nd | nd | 64±12<br>(41) | nd | 27±6<br>(75) | nd | 94±20<br>(15) | AML6=E2 |
| +4<br>w | 73±14 | nd | nd | 25±4<br>(65) | nd | 18±2<br>(75) | nd | 73±14<br>(59) | HiDAC+AMSA=E2 |
| 9 | 40±6 | 16±4<br>(60) | 12±1<br>(69) | 8±3<br>(80) | 14±4<br>(65) | 1±1<br>(99) | 0<br>(100) | nd | AML6=CR, early relapse |
| +8<br>w | 21±4 | 3±1<br>(86) | 1±5<br>(95) | 0<br>(100) | 10±5<br>(52) | 0<br>(100) | 0<br>(100) | nd | HiDAC+AMSA=CR |
| 10 | 54±1 | nd | nd | 56±4<br>(0) | 63±7<br>(0) | 45±12<br>(15) | 6±3<br>(89) | 44±4<br>(17) | AML6=E2 |
| +4<br>w | 55±11 | nd | nd | 3±10<br>(40) | nd | 38±10<br>(31) | nd | 47±15<br>(15) | HiDAC+AMSA=CR |
| 11 | 181±6 | nd | nd | 50±39<br>(72) | 0<br>(100) | 0<br>(100) | nd | 144±7<br>(21) | AML7=E2 |
| +6<br>w | 48±2 | nd | nd | 0<br>(100) | 0<br>(100) | 15±1<br>(68) | nd | 44±6<br>(10) | VP16+AMSA=E2 |
| 12 | 83±6 | nd | nd | 0<br>(100) | nd | 20±1<br>(76) | nd | 49±5<br>(41) | AML8=E2 |
| +3<br>w | 19±2 | nd | nd | 0<br>(100) | nd | 6±2<br>(68) | nd | 10±2<br>(49) | VP16+AMSA=E2 |

w: weeks; PE1: number of colonies/1 x $10^4$ cells plated. CFU-L growth after continuous exposure to $10^{-7}$, $10^{-6}$ and $10^{-5}$M of ara-C or to 30 min exposure to $10^{-7}$, $10^{-6}$ and $10^{-5}$M of DNR. Percentage of CFU-L inhibition is given in brackets.

*Treatments:* TAD: 6-thioguanin, 200 mg m$^{-2}$ x 7, ara-C 200 mg m$^{-2}$ x 7, DNR 60 mg m$^{-2}$ x 3; AML5 of EORTC: adriamycin 50 mg m$^{-2}$ x 1, vincristin 1 mg m$^{-2}$, Ara-C 160 mg m$^{-2}$ x 7. AML6,7,8 of EORTC: DNR 45 mg m$^{-2}$ d1–d3 (30 mg m$^{-2}$ in AML7), vincristin 1.4 mg m$^{-2}$ d2 (AML6,7), ara-C 200 mg m$^{-2}$ d1–d7; HiDAC+AMSA: ara-C 1 or 2 g m$^{-2}$ x 12+AMSA 120 mg m$^{-2}$ x 3 VP16+AMSA=VP16: 100 mg m$^{-2}$ x 5+AMSA 100 mg m$^{-2}$ x 5. E1: Complete resistance with persistence of circulating leukaemic cells without aplasia; E2: failure with leukaemic regrowth; E3: prolonged aplasia with leukaemic regrowth; CR: complete remission.

**Table II** Two determinations of CFU-L first plating efficiency (PE1), *in vitro* sensitivity to ara-C and DNR, treatment received and clinical results of these treatments in patients relapsing after achieving a complete remission.

| | | *Drug exposure to* | | | | | | |
|---|---|---|---|---|---|---|---|---|
| | | *Ara-C* | | | *DNR* | | | |
| *Pts* | *PE1* | $10^{-7}$ M | $10^{-6}$ M | $10^{-5}$ M | $10^{-7}$ M | $10^{-6}$ M | $10^{-5}$ M | *Treatment received* |
| 13 | 22±7 | 65±5 | 21±2 | 18±7 | 14±1 | 0 | 0 | AML6, AMSA+HiDAC/ |
| | | (0) | (5) | (18) | (36) | (100) | (100) | AMSA+5AZA |
| +6 | 29±6 | 56±4 | 28±4 | 19±4 | 32±4 | 18±5 | 5±1 | HiDAC+AMSA=E2 |
| m | | (0) | (0) | (33) | (0) | (37) | (82) | |
| 14 | 16±2 | 6±3 | 3±1 | 5±2 | 5±3 | 1±1 | 0 | AML5, DNR+VCR, |
| | | (62) | (81) | (68) | (68) | (94) | (100) | 6MP±PU |
| +5 | 82±14 | nd | 60±1 | 63±3 | 60±13 | 59±6 | 26±4 | AMSA+HiDAC=E2 |
| m | | | (27) | (22) | (27) | (28) | (68) | |
| 15 | 40±9 | 42±4 | 27±2 | 35±2 | 0 | 0 | 0 | AML6, AMSA+HiDAC/ |
| | | (0) | (31) | (13) | (100) | (100) | (100) | AMSA+5AZA |
| +6 | 53 | nd | 47 | 56 | 60 | 40 | 24 | AML6=E2 |
| m | | | (11) | (0) | (0) | (24) | (55) | |
| 16 | 66±15 | 42±10 | 16±3 | 1±1 | 65±3 | 30±9 | nd | AML6, DNR+Ara-C |
| | | (36) | (76) | (98) | (0) | (54) | | |
| +16 | 73±9 | 27±1 | 26±6 | 27±1 | 33±5 | 25±4 | 6±1 | AML6=CR |
| m | | (63) | (64) | (63) | (55) | (66) | (92) | |
| 17 | 158±30 | 27±4 | 18±5 | 14±6 | 63±6 | 23±5 | nd | AML5, 6TG+ara-C+ |
| | | (82) | (89) | (91) | (61) | (85) | | Immunotherapy |
| +30 | 72±9 | 31±13 | 38±4 | 34±2 | 21±6 | 21±5 | 0 | AML6=E4 |
| m | | (57) | (48) | (53) | (71) | (71) | (100) | |
| 18 | 38±5 | 28±4 | 14±2 | 12±3 | 25±1 | 0 | 0 | AML6, DNR+6MP+MTX |
| | | (26) | (61) | (68) | (34) | (100) | (100) | |
| +11 | 46±2 | 39±2 | 26±6 | 26±5 | 18±6 | 31±5 | 0 | HiDAC+AMSA=CR |
| m | | (15) | (44) | (43) | (61) | (33) | (100) | |
| 19 | 24±4 | nd | 13 | 6 | nd | 13 | nd | AML6, AMSA+HiDAC/ |
| | | | (46) | (75) | | (46) | | AMSA+5AZA |
| +10 | 114±3 | 121±19 | 57±15 | 39±2 | 95±9 | 51±2 | 0 | HiDAC+AMSA=E4 |
| m | | (0) | (50) | (66) | (16) | (55) | (100) | |
| 20 | 46±5 | 60±6 | 58±8 | 27±1 | 22±1 | 0 | 0 | AML6, DNR+ara-C |
| | | (0) | (0) | (41) | (52) | (100) | (100) | |
| +20 | 92±8 | 42±5 | 27±3 | 9±6 | 23±3 | 11±3 | 0 | HiDAC+AMSA=CR |
| m | | (54) | (71) | (90) | (75) | (88) | (100) | |
| 21 | 40±13 | 48±11 | 19±1 | 12±4 | 55±9 | 29±4 | 20±5 | AML6, DNR+ara-C |
| | | (0) | (53) | (70) | (37) | (27) | (50) | |
| +14 | 51±2 | nd | nd | nd | 52±4 | 16±1 | 1±1 | AML6=CR |
| m | | | | | (0) | (69) | (97) | |
| 22 | 56±22 | 25 | 16 | 0 | nd | 13±2 | 0 | AML6, HiDAC+AMSA |
| | | (55) | (71) | (100) | | (22) | (100) | |
| +10 | 81±9 | nd | nd | 48±4 | 82±10 | 43±3 | 0 | AML6=CR |
| m | | | | (41) | (0) | (47) | (100) | |
| 23 | 23±4 | nd | 42±14 | 7±3 | nd | nd | nd | AML6, AMSA+HiDAC/ |
| | | | (0) | (69) | | | | AMSA+5AZA |
| +17 | 54±3 | nd | nd | 48±3 | nd | 35±4 | nd | AML8=CR |
| m | | | | (11) | | (34) | | |

m: months; PE1: number of colonies/$1\times10^4$ cells plated. CFU-L growth after continuous exposure to $10^{-7}$, $10^{-6}$ and $10^{-5}$ M of ara-C or to 30 min exposure to $10^{-7}$, $10^{-6}$ and $10^{-5}$ M of DNR. Percentage of CFU-L inhibition is given in brackets.

*Treatments:* AML5 of EORTC: adriamycin 50 mg m$^{-2}\times 1$, vincristin 1 mg m$^{-2}$, ara-C 160 mg m$^{-2}\times 7$; consolidation: *idem*, maintenance: either purinethol + 6-mercaptopurine + (DNR + vincristin × 9) + androgen or 6-thioguanin + ara-C immunotherapy. AML6,8 of EORTC: DNR 45 mg m$^{-2}$ d1–d3, vincristin 1.4 mg m$^{-2}$ d2 (AML6), ara-C 200 g m$^{-2}$ d1–d7; consolidation in AML6: *idem* but DNR d1 only. Maintenance for AML6: 6 courses of either DNR + ara-C or AMSA + HiDAC/AMSA + 5 AZA. AMSA + HidDAC: AMSA 120 mg m$^{-2}\times 5$ + ara-C 3 g m$^{-2}\times 4$. HiDAC + AMSA: ara-C 1 or 2 g m$^{-2}\times 12$ + AMSA 120 mg m$^{-2}\times 3$; E2: failure with leukaemic regrowth; E4: toxic death; CR: complete remission.

In our patients, *in vitro* CFU-L sensitivity to $10^{-6}$ M DNR was higher in patients entered into CR (73±22%) than in resistant patients (55±41%), but without statistical significance.

### Discussion

Repeated study of CFU-L drug sensitivity in 23 patients treated for AML showed different results according to the time of the second study.

In treatment failures, the sensitivity to DNR concentrations used for *in vitro–in vivo* correlations did not change significantly when tested before and after chemotherapy. In these resistant patients, the interval between the *in vitro* studies was short (6 weeks), a time probably insufficient to develop a leukaemic clone with properties different than those observed before therapy. These results confirm the preliminary report of McCulloch (1982), who have repeatedly assessed the self renewal capacity and drug sensitivity of circulating CFU-L: these parameters showed little variation, except evidence of developing drug resistance in a few patients (one relapse and one primary resistance). These data suggested that drug sensitivity and self renewal capacity are heritable characteristics in leukaemic clones, but the small number of patients (7) does not permit definitive conclusions.

Treatment failure was predicted by the first *in vitro* CFU-L sensitivities to DNR but not to ara-C. This superior predictive value of DNR sensitivity was also found in a larger series (Marie *et al*., 1987).

In patients achieving a complete remission, the emergence of a leukamic clone with different properties from that observed at diagnosis was noted at relapse in a majority of cases: the sensitivity to $10^{-6}$M DNR or $10^{-5}$M ara-C decreased markedly in 5 patients, increased in one and remained stable in the five others. This can be related to other modifications observed in the study of leukaemic populations in relapse *viz*. additional chromosomal abnormalities (Pui *et al*., 1986), or change in surface phenotype (Borella *et al*., 1979; Lauer *et al*., 1982; Pui *et al*., 1986; Stass *et al*., 1984), suggestive of clonal evolution. Schwarzmeier *et al*. (1984), using a short term test with $^3$H uridine incorporation in a whole peripheral blast cell population, also noted an *in vitro* acquired drug resistance in 2 patients tested repeatedly.

The increase of CFU-L drug resistance observed in several relapsing patients could explain the poor prognosis of AML patients in relapse. The reduction of leukaemic cells during induction treatment and the long time between the two *in vitro* studies in these patients are in favour of the expansion of a subclone either existing from the beginning of the disease, or selected by mutations in the residual leukaemic clone.

In the great majority of cases, the second CR is shorter than the first one, like in our present study, reflecting a relative chemoresistance, with only moderate cytoreduction during reinduction treatment. Our comparative observations in resistant and relapsing patients are surprising, if one takes into account the good correlation normally observed between *in vitro* sensitivity of clonogenic cells to DNR+ara-C and clinical response (Zittoun *et al*., 1987): one could expect, on the contrary, the emergence of *in vitro* resisting clones after failure of induction treatment and persistence of sensitive clones in most relapsing patients. This could be explained either by the inability of our clonogenic assay to measure leukaemic stem cell properties, or by other resistance mechanisms involving cell kinetic (Raza *et al*., 1987) or pharmacological factors (Plunkett *et al*. 1985). The properties of more primitive leukaemic stem cells could be studied in liquid cultures (Nara & McCulloch, 1985), and could perhaps better account for the clinical evolution of the disease.

This work was supported in part by grants from 'La Ligue Française contre le Cancer' and Université Paris VI.

### References

BORELLA, L., CASPER, J.T. & LAUER, S.J. (1979). Shifts in expression of cell membrane phenotypes in childhood lymphoid malignancies at relapse. *Blood*, **54**, 64.

HERZIG, R.H., WOLFF, S.N., LAZARUS, H.M., PHILLIPS, G.L., KARANAS, C. & HERZIG, G.P. (1983). High dose cytosine arbinoside therapy for refractory leukemia. *Blood*, **62**, 361.

LAUER, S., PIASKOWSKI, V., CAMITTA, B. & CASPER, J. (1982). Bone marrow and extramedullary variations of cell membrane antigen expression in childhood lymphoid neoplasia at relapse. *Leuk. Res*., **6**, 769.

McCULLOCH, E.A., CURTIS, J.E., MESSNER, H.A. & SENN, J.S. (1981). The heritable nature of clonal characteristics in acute myeloblastic leukemia. *Blood*, **58**, 105.

McCULLOCH, E.A., CURTIS, J.E., MESSNER, H.A., SEEN, J.S. & GERMANSON, T.P. (1982). The contribution of blast cell properties to outcome variation in acute myeloblastic leukemia. *Blood*, **59**, 601.

MARIE, J.P., ZITTOUN, R., THEVENIN, D., MATHIEU, M. & VIGUIE, F. (1982). *In vitro* culture of clonogenic leukemic cells in acute myeloid leukemia: growth pattern and drug sensitivity. *Br. J. Haematol*., **55**, 427.

MARIE, J.P., ZITTOUN, R., DELMER, A. & THEVENIN. D. (1987). Prognostic value of clonogenic assay for induction and duration of complete remission in acute myeloblastic leukemia. *Leukemia*, **1**, 121.

MARIE, J.P. (1987). Cultures de cellules souches clonogènes dans les leucémies aigues myéloides humaines: interet pratique. *Presse Med*., **16**, 2059.

MINDEN, M.D., BUICK, R.N. & McCULLOCH, E.A. (1979). Separation of blast cells and T lymphocyte progenitors in the blood in patients with acute myeloid leukemia. *Blood*, **54**, 186.

NARA, N. & McCULLOCH, E.A. (1985). The proliferation in suspension of the progenitors of the blast cells in acute myeloid leukemia. *Blood*, **65**, 1484.

PETERSON, B.A. & BLOOMFIELD, C.D. (1981). Re-induction of complete remission in adults with acute non-lymphocytic leukemia. *Leuk. Res*., **5**, 81.

PLUNKETT, W., IACOBINS, S., ESTEY, E., DANHAUSER, C., LILIEMARK, J.O. & KEATING, M.J. (1985). Pharmacologically directed Cytosine Arabinoside therapy for refractory leukemia. *Semin. Oncol*., **12**, Suppl. 3: 20.

PREISLER, H.D. (1980). Prediction of response to chemotherapy in acute myelocytic leukemia. *Blood*, **56**, 361.

PUI, C.H., RAIMONDI, S.C., BEHM, F.G. & 6 others (1986). Shifts in blast cell phenotype and karyotype at relapse of childhood lymphoblastic leukemia. *Blood*, **68**, 1306.

RAZA, A., MAHESHWARI, Y., MANDAVAN, N. & 11 others (1987). Cell cycle and drug sensitivity studies of leukemic cells that appear relevant in determining response to chemotherapy in acute myeloid leukemia. *Semin. Oncol*., **14**, Suppl. 1: 217.

SCHWARZMEIER, J.D., PAIETTA, E., MITTERMAYER, K. & PIERKER, R. (1984). Prediction of the response to chemotherapy in acute leukemia by a short term test *in vitro*. *Cancer*, **53**, 390.

SKIPPER, H.E., SCHABEL, F.M. & LLOYD, H.H. (1978). Experimental therapeutics and kinetics: selection and overgrowth of specifically and permanently drug resistant tumor cells, In *Leukemia and Lymphoma*, (eds) Freireich *et al*., Grune ë Stratton, New York, p. 342.

STASS, S., MIRRO, J., MELVIN, S., PUI, C.H., MURPHY, S.B. & WILLIAMS, D. (1984). Lineage switch in acute leukemia. *Blood*, **64**, 701.

ZITTOUN, R., MARIE, J.P., BRILHANTE, D. & DELMER, A. (1987). Prediction of induction and duration of complete remission in acute myeloid leukemia: value of clonogenic cell properties, In *Haematology and Blood Transfusion*, (eds) Büchner *et al*., Springer-Verlag, Berlin, **30**, 45.